# Supplementary material for: MetaRibo-Seq measures translation in microbiomes
Source: Nat Commun. 2020 Jun 29;11:3268. doi: 10.1038/s41467-020-17081-z (PMC7324362; doi:10.1038/s41467-020-17081-z)
Supplement: Supplementary file 10 — Supplementary Data 7 [file 41467_2020_17081_MOESM10_ESM.zip › File2/Confidence_VeryHigh_Taxonomy/65816_out.krona.html]

Javascript must be enabled to view this page.

members
magnitude
magnitudeUnassigned
count
unassigned
taxon
rank

65816\_out

4

4
2
superkingdom

976
phylum
4

class
200643
4

4
171549
order

4
family
815

4
genus
816


SRS019968\_contig\_number\_68SRS057478\_contig\_number\_492
2
28116
species

species
47678
1

SRS893230\_contig\_number\_686

1
species
338188

SRS144753\_contig\_number\_5460
